# Development of a DIY rehabilitation device for lower limb weakness in acute to subacute ischemic stroke

**DOI:** 10.1016/j.mex.2021.101582

**Published:** 2021-11-25

**Authors:** Noppamad Tangmanee, Sombat Muengtaweepongsa, Wiroj Limtrakarn

**Affiliations:** a Medical Engineering Program, Faculty of Engineering, Thammasat University, Pathum Thani 12120, Thailand; b Excellence Center in Stroke, Department of Internal Medicine, Faculty of Medicine, Thammasat University, Pathum Thani 12120, Thailand; c Department of Mechanical Engineering, Faculty of Engineering, Thammasat University, Pathum Thani 12120, Thailand

**Keywords:** Do-It-Yourself (DIY), Medical device, Home rehabilitation, Stroke rehabilitation, Leg weakness

## Abstract

Many patients have significantly lower limb weakness after getting a stroke. Continuous regular physical therapy is essential to promote the improvement of the weakness and overall outcomes. Home rehabilitation provides motivation and enhances regular rehabilitation in stroke patients. The Do-It-Yourself (DIY) medical device is developed to fill the gap of unmet medical management needs and becomes increasingly applied in rehabilitation. The DIY device should support the concept of home rehabilitation in stroke patients. We designed and developed a low-cost, easy-to-use, DIY rehabilitation device to promote regular physical therapy in stroke patients with lower limb weakness. The methods and rationale of device development were described. The feasibility and safety of the device were also evaluated.

• The DIY rehabilitation device for the lower limb is convenient and easy to assembly.
• Regular home rehabilitation is enhanced with the DIY rehabilitation device.
• The device is feasible and safe for physical therapy in stroke patients with lower limb weakness.

## Graphical abstract

**Figure:**
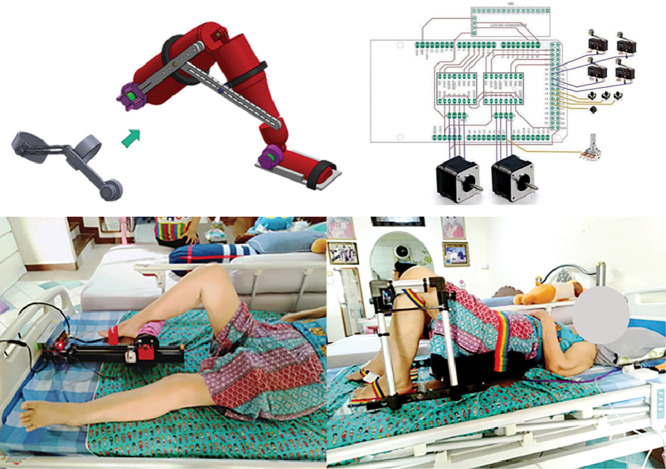

Specifications table

**Table:** 

Subject area:	Medicine and dentistry
More specific subject area:	*Rehabilitation*
Method name:	*Semi-assisted robotic rehabilitation device for lower limb weakness*
Name and reference of original method:	*N/A*
Resource availability:	*N/A*

*Method details *[Methodological protocols should be in sufficient detail to be replicated. There is no word limit! You can include figures, tables, videos – anything that you feel will help others to reproduce the method. The main focus of the paper should be on the technical steps required for this method, more than results; where appropriate, guide the reader through the procedure and provide all extra observations or ”tricks” alongside the protocol. Results and Discussion are not sections included in the MethodsX format. However, providing data that validate the method is valuable and required. This section could become a “method validation” paragraph within the Method Details section.]*

## Introduction

Stroke is a central nervous system disorder that causes the sudden onset of neurologic manifestations. Dysfunction of blood vessels causes corresponding brain lesions in their feeding area. It is categorized into two main subgroups according to its pathogenesis: ischemic stroke and hemorrhagic stroke. The prevalence of ischemic stroke cases is approximately 85% of all stroke patients, and the remaining 15% are intracranial hemorrhages [1]. Disability is a common condition in acute ischemic stroke patients despite standard treatment [2]. As a result, stroke is the leading cause of disability in adults [3]. Thirty-five percent of stroke patients survived with a significant weakness in the legs. They could not regain their lower limb strength to function, and 25 percent needed help in walking [4]. In patients with acute stroke beginning with complete leg paralysis, only 10 percent of them could return to walking without assistance [5].

Physical therapy (PT) is an essential treatment for stroke patients. This therapy stimulates the recovery of the motor system of the limbs [6]. Several training methods of PT are available, including constraint-induced movement therapy (CIMT), robotic-assisted rehabilitation, virtual reality, and functional electrical stimulation (FES) [7]. Although all methods should help improve weakness in stroke patients, each method's essential keys to success are still regular performance [8]. Some patients were unable to continue rehabilitation due to insufficient physical therapy medical equipment. There is still a matter of physical therapy techniques. In some hospitals, there are insufficient physical therapists for the number of patients. Also, when the patient returns home, he or she has to continue with physical therapy. Some people still have to return to rehab at the hospital. Due to the need to use physical therapy tools, patients have to pay their money to travel to the hospital. Some poor people from remote areas cannot afford the expense of traveling. As a result, patients with these problems do not have physical therapy regularly. Muscle weakness increases. The joints are stuck. Chronic paralysis puts the patient into a bed-bound condition [9]. However, if a device or method encourages the patient to exercise regularly at home or the nearby community hospital, it will reduce costs.

This research project is designed to develop mechanisms that can help restore lower limb muscle strength. We aim to design a device to assist in physical therapy and prevent the recession of lower limb muscles due to muscle weakness and prolonged bed-bound patients with acute stroke. Therefore, we are interested in machine or robotic rehabilitation methods to perform lower limb muscle rehab since in bed. Considering the speed of recovery, tool performance, ease of use should help keep the muscles power, and the patient returns to a routine or the most normal equivalent. We also conducted a comparison study between experimental (lower limb rehabilitation machine) and control (standard treatment) groups for the feasibility of the device use.

## Methods

We conducted two steps of developing a DIY Rehabilitation Machine consisting of the prototypes of lower limb rehabilitation machines and the generated machines to collect clinical data.

**Fig. 1:**
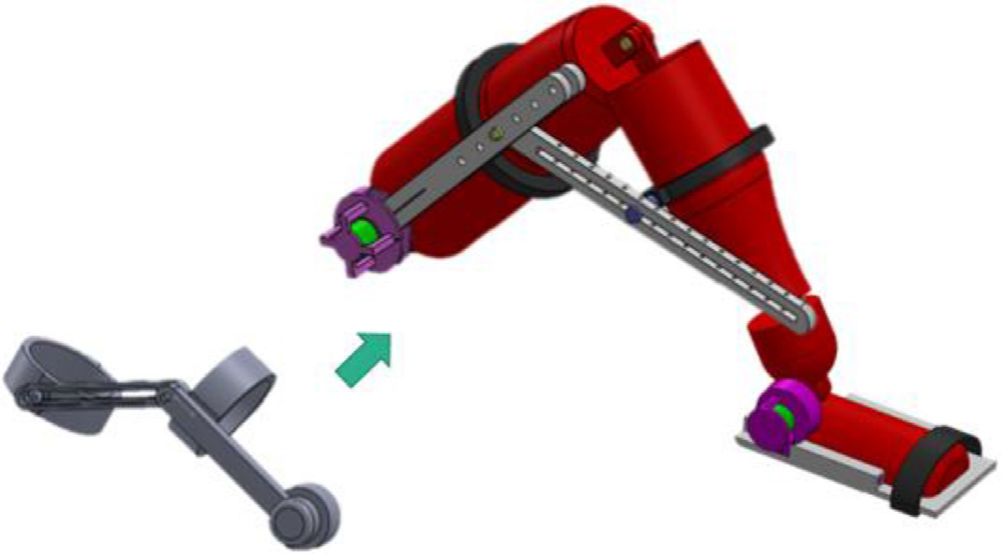
Design model by Solid Works; can be used for either left or right lower limb.
1.1.2. We calculated motor power and designed a regeneration operation circuit of lower limb muscles. The force was calculated from the three lower limb movement patterns, which consisted hip-knee flexion-extension, (2) hip lifting (bridging), and (3) ankle dorsiflexion-plantarflexion of (1). We calculated by determining the extent to which the patient's weight is up to 80 kg and a height of approximately 170 cm. We calculated the force at different lengths and weights by the equation as follows:
  F=(%bodyweight)x(bodymass)x(gravitationalacceleration)
  $$F_{\text{femur}}\;=\left(0.1447\right)x\left(80\;\text{kg}.\right)x\left(9.81m/s^{2}\right)\;=\;113.5606\;N.$$
  $$F_{\text{trunk}}\;=\left(0.4302\right)x\left(80\;\text{kg}.\right)x\left(9.81\;m/s^{2}\right)\;=\;337.621N.$$
  $$F_{\text{tibia}}\;=\left(0.0457\right)x\left(80\;\text{kg}.\right)x\left(9.81\;m/s^{2}\right)\;=\;35.8654\;N.$$
  $$F_{\text{foot}}\;=\left(0.0133\right)x\left(80\;\text{kg}.\right)x\left(9.81\;m/s^{2}\right)\;=\;10.4378\;N.$$

1. Create a prototype of the lower limb rehabilitation machine.
1.1. We studied literature review, related research, clinical features, and physical therapy using various stroke treatment methods.
1.1.1. We designed the device by SolidWorks program based on the lower limb movement, including hip lifting-up, hip flexion-extension, knee flexion-extension, and ankle dorsiflexion-plantarflexion as shown in Fig. 1.

According to the following three exercises, we calculated the electric motor (EM) power used to construct the device based on the force mentioned above.

**Fig. 2:**
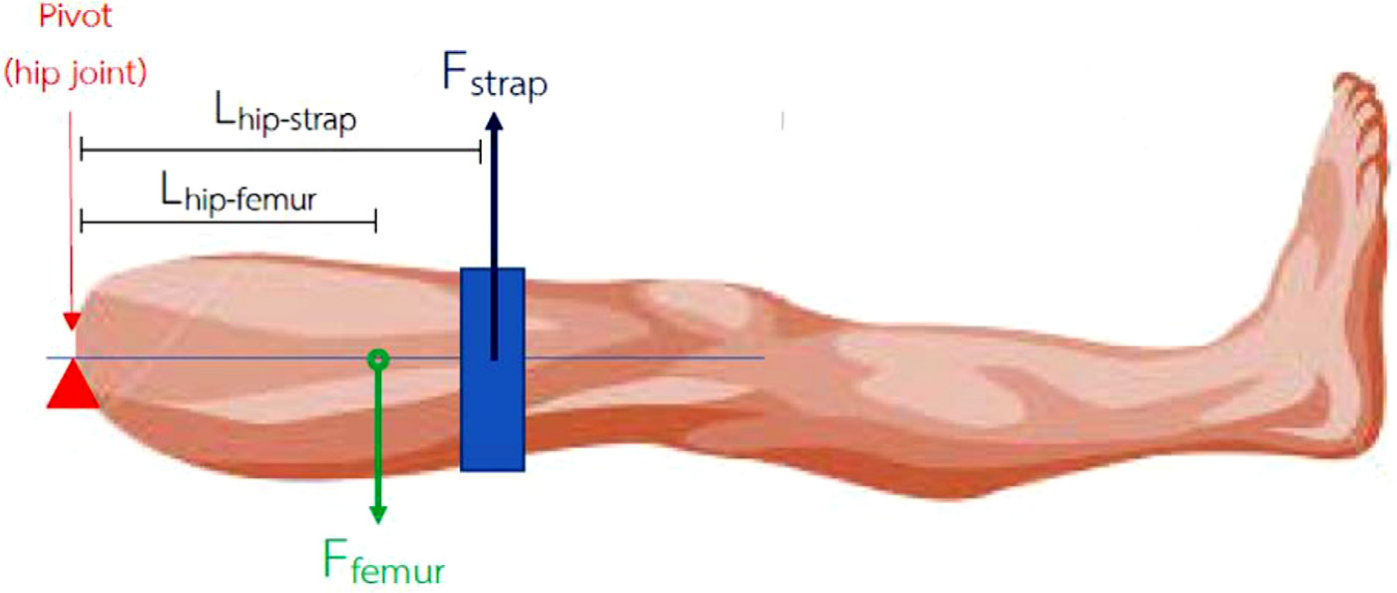
Free body diagram of patient's leg during knee flexion and extension exercise.

**Fig. 3:**
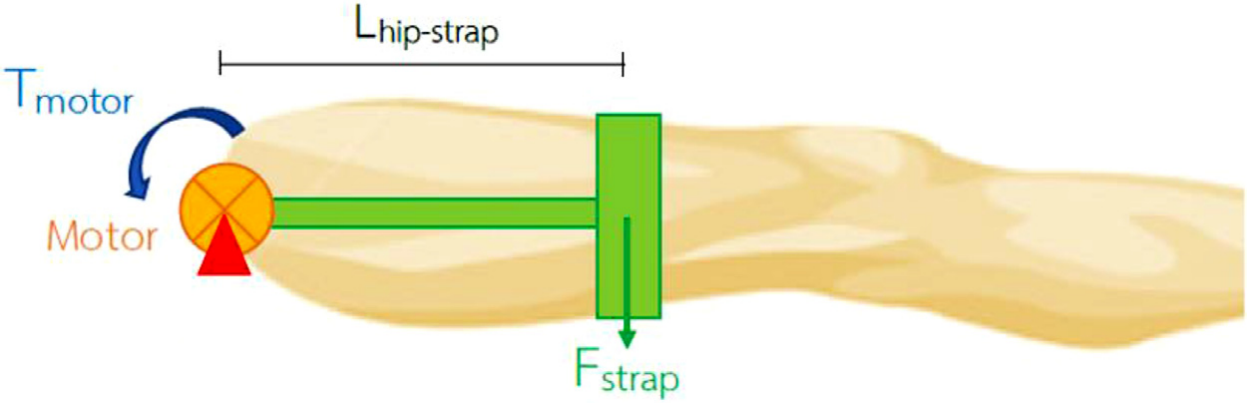
Free body diagram of exoskeleton during knee flexion and extension exercise.

(1) hip-knee flexion-extension (Fig. 2, Fig. 3)
  ΣM=0
  $$\left(F_{\text{strap}}\right)\left(L_{\text{hip}\_\text{strap}}\right)-\left(F_{\text{femur}}\right)\left(L_{\text{hip}\_\text{femur}}\right)=0$$
  $$\left(F_{\text{strap}}\right)\left(0.23\right)-\left(113.5606\right)\left(0.1752\right)=0$$
  $$\left(F_{\text{strap}}\right)\left(0.23\right)=19.8958$$
  $$F_{\text{strap}}=86.5036\;N.$$

$$\begin{array}{ccc} \tau & = & F\;x\;r\\ \tau_{\text{motor}} & = & \left(\text{Fstrap}\right)\left(\text{Lhip}-\text{strap}\right)\\ \tau_{\text{motor}} & = & \left(86.5036\right)\left(0.23\right)\\ \tau_{\text{motor}} & = & \;19.8958\;N-m. \end{array}$$

Use safety factor (N_s_) of 1.5 to account for friction, therefore

$$\begin{array}{ccc} \tau_{\text{motor}} & = & \;19.8958\;N-m.\;X\;1.5\\ \tau_{\text{motor}} & = & \;29.8437\;N-m. \end{array}$$

Therefore, we needed an EM with a torque of about 30 N-m.

(2) hip lifting (Bridging)

**Fig. 4:**
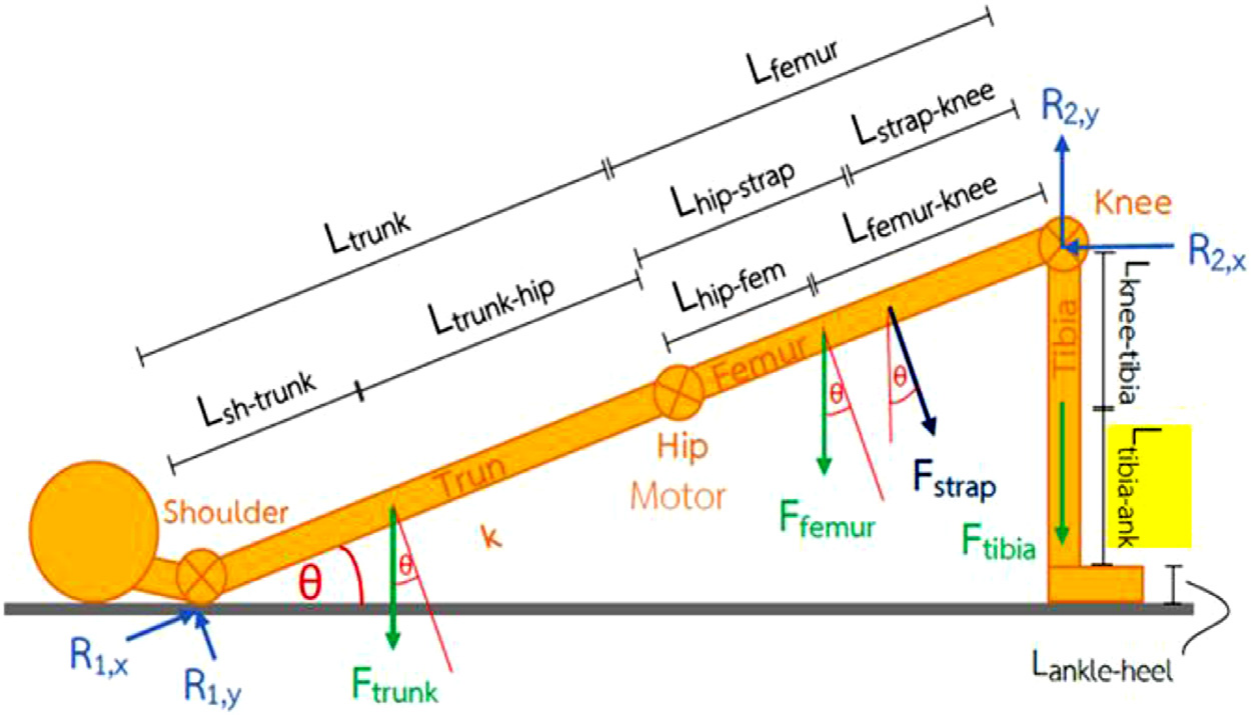
Free body diagram of patient's leg during bridging exercise.

Calculate the angle at which the hips are raised. θ (Fig. 4):

$$\theta =\sin^{-1}\left(\frac{0.4323}{1.045}\right)$$

$$\theta =24.4365^{\circ }$$

To calculate the moment:

(1) $$\begin{array}{ccc} \Sigma MR_{1} & = & 0-0.825F_{\text{strap}}+R_{2,x}\left(\sin\theta \right)\left(1.045\right)+R_{2,y}\left(\cos\theta \right)\left(1.045\right)=\left(F_{\text{trunk}}\right)\left(\cos\theta \right)\left(0.2407\right)\\ & & +\;F_{\text{femur}}\left(\cos\theta \right)\left(0.7702\right)+F_{\text{tibia}}\left(0.9514\right)-0.825F_{\text{strap}}+0.4323R_{2,x}+0.9514R_{2,y}=187.5511 \end{array}$$

(2) $$\begin{array}{ccc} \Sigma M_{\text{strap}} & = & 0-0.825R_{1,y}+R_{2,x}\left(\sin\theta \right)\left(0.22\right)+R_{2,y}\left(\cos\theta \right)\left(0.22\right)+F_{\text{trunk}}\left(\cos\theta \right)\left(0.5843\right)\\ & & +\;F_{\text{femur}}\left(\cos\theta \right)\left(0.0548\right)-F_{\text{tibia}}\left(\cos\theta \right)\left(0.22\right)\;=\;0-0.825R_{1,y}+0.091R_{2,y}=-178.2682 \end{array}$$

(3) $$\Sigma MR_{2}=00.22F_{\text{strap}}-1.045R_{1,y}+\left(F_{\text{trunk}}\right)\left(\cos\theta \right)\left(0.8043\right)+F_{\text{femur}}\left(\cos\theta \right)\left(0.2794\right)=00.22F_{\text{strap}}-1.045R_{1,y}=-275.821$$

(4) $$\begin{array}{ccc} \Sigma F_{y} & = & 0-F_{\text{strap}}\left(\cos\theta \right)+R_{1,x}\left(\sin\theta \right)+R_{1,y}\left(\cos\theta \right)+R_{2,y}=F_{\text{trunk}}+F_{\text{femur}}\\ & & +\;F_{\text{tibia}}-0.9104F_{\text{strap}}+0.4137R_{1,x}+0.9104R_{1,y}+R_{2,y}=487.0469 \end{array}$$

(5) $$\begin{array}{ccc} \Sigma F_{x} & = & 0F_{\text{strap}}\left(\sin\theta \right)+R_{1,x}\left(\cos\theta \right)-R_{1,y}\left(\sin\theta \right)\\ & & -\;R_{2.x}=00.4137F_{\text{strap}}+0.9104R_{1,x}-0.4137R_{1,y}-R_{2.x}=0 \end{array}$$

Solve Eq. (1) –through (5), yields

$$F_{\text{strap}}=-232.1225\;N$$

$$R_{1,x}=199.2551\;N$$

$$R_{1,y}=215.0756\;N$$

$$R_{2,x}=-3.604\;N$$

$$R_{2,y}=-2.5141\;N$$

τ=Fxr

$$\tau_{\text{motor}}=\left(\text{Fstrap}\right)\left(\text{Lhip}-\text{strap}\right)$$

$$\tau_{\text{motor}}=\left(232.1225\right)\left(0.23\right)$$

$$\tau_{\text{motor}}=\;53.3882\;N-m$$

Use safety factor (N_s_) of 1.5 times a calculated torque to achieve friction forces in the system, then

$$\tau_{\text{motor}}=\;53.3882\;N-m\;\times \;1.5$$

$$\tau_{\text{motor}}=\;80.0823\;N-m$$

Therefore, we needed an EM with a torque of about 81 N-m.

**Fig. 5:**
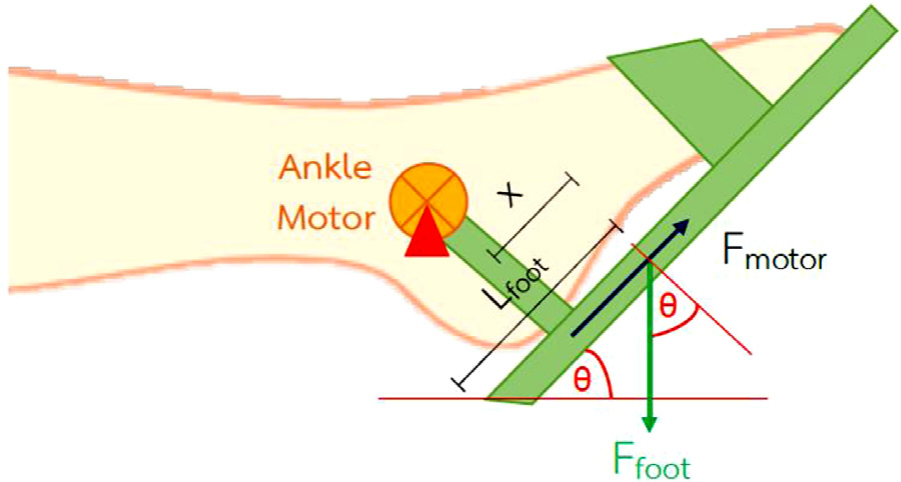
Free body diagram of patient's foot during ankle flexion and extension.

(3) ankle dorsiflexion-plantarflexion (Fig. 5)

Assume the patient's foot drop about 30 degrees while lying on the bed. Therefore, the angle between the ankle is perpendicular to the foot drop.

$$\theta =90^{\circ }-\emptyset \;=90^{\circ }-30^{\circ }$$

$$\theta =60^{\circ }$$

$$\text{Let}\;\;x\;\;=L_{\text{foot}}/2=0.0633\;\;m.$$

ΣM=0

$$\left(F_{\text{motor}}\right)\left(l_{\text{ankle}\_\text{heel}}\right)-\left(F_{\text{foot}}\right)\left(\cos\theta \right)\left(x\right)=0$$

$$0.0723\left(F_{\text{motor}}\right)=10.4378\cos60^{\circ }\left(0.0633\right)$$

$$\left(F_{\text{motor}}\right)=4.5692\;N.$$

τ=Fxr

$$\tau_{\text{motor},2}=\left(F_{\text{motor}}\right)\left(l_{\text{ankle}\_\text{heel}}\right)$$

$$\tau_{\text{motor},2}=\left(84.5692\right)\left(0.07230.23\right)$$

$$\tau_{\text{motor},2}=\left(0.3304\right)\;N-m.$$

Use safety factor (N_s_) of 1.5 times a calculated torque to achieve friction forces in the system, therefore

$$\tau_{\text{motor},2}=\left(0.3304\right)\;N-m.\;x\;1.5$$

$$\tau_{\text{motor},2}\;=\;0.4955\;N-m.$$

Therefore, we needed an EM with a torque of 0.5 N-m.

1.1.1. The circuit of the lower limb rehabilitation machine operation was shown in Fig. 6.

**Fig. 6:**
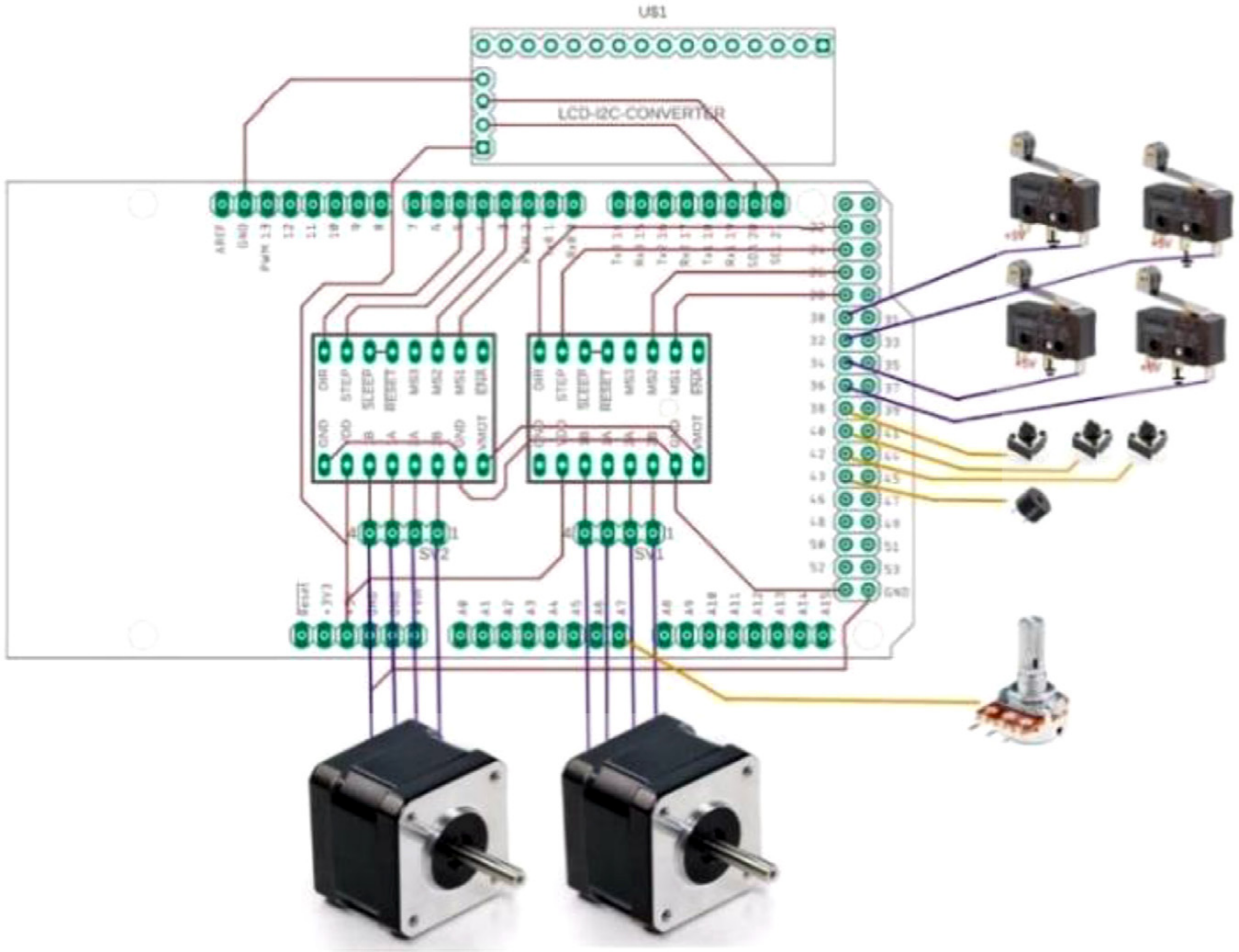
Control circuit of the lower limb rehabilitation machine.
1.2. We recommended not to apply to patients who have complete sensation loss. It can cause bruises and heel injuries when bending the leg.
2. Clinical data collection methods

It is experimental research. We aimed to test a prototype device in acute stroke patients admitted to Thammasat University Hospital.

**Fig. 7:**
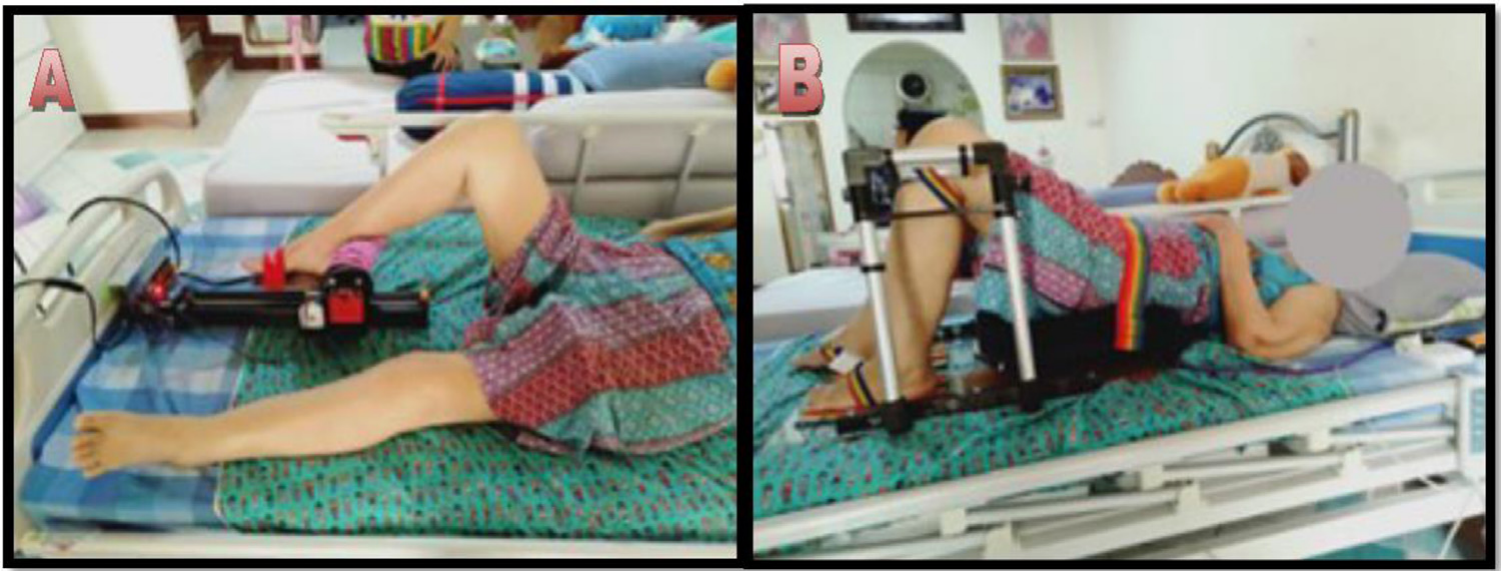
A. hip flexion-extension, knee flexion-extension, ankle dorsiflexion-plantarflexion. B. hip bridging.
2.3.3.2. Patients in the control group got the standard rehabilitation for 30 minutes and focus exercise: hip flexion-extension, knee flexion-extension, ankle dorsiflexion-plantarflexion and hip bridging 30 minutes, five days a week for three months.
2.3.3.3. Hip flexion-extension, knee flexion-extension range of motion (ROM) 0˚- 80˚, ankle dorsiflexion-plantarflexion ROM 0˚- 60˚ and hip bridging: lift height 15 cm.
2.3.4. Outcomes measurement
2.3.4.1. We measured and compared the Fugl-Meyer Assessment for lower extremity (FMA-LE) and Brunstrom Stage of Motor Recovery of the lower extremity and modified Rankin Scale (mRS) before treatment, after two, four, eight, and twelve weeks of treatment.
2.3.5. Statistical analysis
2.3.5.1. We compared the mean and standard deviation of age, days post stroke onset, degree of weakness, and individual NIHSS scores between the experimental and control groups.
2.3.5.2. We analyzed one-way variation (One-way repeated measures ANOVA) comparing the FMA-LE and Brunstrom Stage of Motor Recovery of the lower extremity and modified Rankin Scale before the trials, weeks 2, 4, 8, and 12.
2.3.5.3. We used paired t-test to compare the values of the FMA-LE and Brunstrom Stage of Motor Recovery of the lower extremity and modified Rankin Scale before treatment and after twelve weeks of treatment.
2.3.5.4. We set the level of statistical significance at 0.05.
2.4. Results
2.4.1. The experimental group had a mean age of 53.8 years and had a mean time from stroke onset of 3.9 days. Among ten in the experimental group, eight people had left hemiparesis. The mean NIHSS score was 11.58 points, and the mean Brunnstrom score was 2.5.
2.4.2. The control group had a mean age of 59.2 years and had a mean time from stroke onset of 4.1 days. Among ten in the control group, nine people had left hemiparesis. The mean NIHSS score was 10.33, and the mean Brunnstrom score was 2.5.

2.1. Population
2.1.1. The population used in this study was a patient who got admitted to the Stroke Unit, Thammasat University Hospital. Both genders who agreed to participate in research projects were eligible. The Thammasat University Ethics Committee approved the protocol (No. MTU-IC-IM-1-102/61 on May 24th, 2018). The Faculty of Medicine, Thammasat University, granted funding for the study in 2018.
2.2. Samples
2.2.1. The sample size was calculated with n4Studies [10], as shown below, using Zakharov et al. [11] by the mean FMA-LE of 11.5 ± 0.84 and 12.69 ± 1.2 in control and experimental groups, a significance level of 0.05 and powers of 0.8. The required sample size was calculated as 24 participants (12 subjects in each group).
  $$n1=\frac{{\left(z_{1}-\frac{\alpha }{2}+z_{1}-\beta \right)}^{2}\left[\sigma_{1}^{2}+\frac{\sigma_{2}^{2}}{r}\right]}{\Delta^{2}}$$
  $$r=\frac{n_{2}}{n_{1}},\;\Delta =\mu_{1-}\mu_{2}$$
2.2.2. Two patients in each group could not complete the study and needed to be excluded. The samples consisted of 20 acute stroke patients who met the inclusion and completed the 12-weeks trial. We divided patients equally by a randomized computer program into an experimental and control group.
2.3. Inclusion / Exclusion criteria
2.3.1 Inclusion criteria
2.3.1.1. An acute ischemic stroke within three months after the onset without any recurrent stroke.
2.3.1.2. Age between 40 and 80 years old.
2.3.1.3. Thai Minimental Status Examination (TMSE) score of more than 22.
2.3.1.4. National Institutes of Health Stroke Scale (NIHSS) between 5 and 15.
2.3.1.5. Brunstrom Stage of Motor Recovery of the lower extremity of more than 1.
2.3.1.6. A patient or relatives can consent to study according to the Human Research Ethics Committee's regulations.
2.3.2. Exclusion criteria
2.3.2.1. A patient had a significant communication defect.
2.3.2.2. Limb weakness not caused by stroke: Parkinson's disease, spinal cord injury, epilepsy, weakness from a head injury, brain tumor.
2.3.2.3. A patient with heart disease, lung disease, joint deformity, joint inflammation.
2.3.2.4. Impaired vision (visual scale in NIHSS = 3) or severe sensory impairment (sensory scale in NIHSS = 2)
2.3.3. Rehabilitation protocol
2.3.3.1. Patients in the experimental group got the standard rehabilitation for 30 minutes following 30 minutes of the exercise with our prototype device (Fig. 7). This session would get done five days a week for three months.

The mean and standard deviation in both groups were not statistically significant. Table 1 showed the demographic data of both groups.

2.4.3. The mean values of FMA-LE and Brunstrom Stage of Motor Recovery (Lower extremity) before the trials, weeks 2, 4, 8, and 12 of the experimental and control groups, were getting a statistically significant improvement. However, the modified Rankin Scale before the trials, weeks 2, 4, 8, and 12 of the experimental and control groups, was not statistically improved.

**Table 1:** Demographic data (Mean±SD) of two groups

Demographic characteristics	Experimental group (n = 10)	Control group (n = 10)	p-value
Age (years)	53.8 ± 6.14	59.2 ± 2.28	0.58
Gender			
male	6	7	
female	4	3	
Paretic side			
left	8	9	0.15
right	2	1	
Time from onset (days)	3.9 ± 1.38	4.1 ± 1.28	0.43
NIHSS	11.58 ± 3.42	10.33 ± 2.92	0.68
Brunnstrom stage	2.50 ± 0.53	2.50 ± 0.53	0.60

NISS = National Institute of Health Stroke Scale

Table 2 showed FMA-LE, Brunstrom Stage of Motor Recovery (Lower extremity), and modified Rankin scale before the trials, weeks 2, 4, 8, and 12 of both groups.

2.4.4. The FMA-LE, Brunstrom Stage of Motor Recovery (Lower extremity), and modified Rankin scale were getting statistically significant improvement when comparing between before and after 12 weeks of treatment in both groups (as shown in Table 3).

**Table 3:** Outcome measure (Means±SD) compared the Fugl-Meyer Assessment for lower extremity (FMA-LE), Brunstrom Stage of Motor Recovery (Lower extremity), and modified Rankin scale before treatment and after treatment of two group.

Outcome measure	Group	Before treatment	After treatment (Week 12)	p-value
FMA-LE	Experimental	14.50 ± 1.58	30.10 ± 1.79	0.03*
	Control	13.60 ± 1.26	27.40 ± 1.89	0.01*
Brunstrom Stage	Experimental	2.50 ± 0.53	5.60 ± 0.52	0.01*
	Control	2.50 ± 0.53	4.60 ± 0.72	0.02*
Modified Rankin Scale	Experimental	4.00 ± 0.52	2.20 ± 0.70	0.02*
	Control	4.10 ± 0.12	2.60 ± 0.31	0.00*

⁎ Significant difference in gains, p<0.05

**Table 2:** Outcome measure (Means±SD) compared the Fugl-Meyer Assessment for lower extremity (FMA-LE), Brunstrom Stage of Motor Recovery (Lower extremity), and modified Rankin scale before treament, weeks 2, 4, 8, and 12 of both groups.

Outcome measure	Week	Experimental	Control	p-value
**FMA-LE**	**Before treatment**	14.50 ± 1.58	13.60 ± 1.26	
	**2**	17.40 ± 1.71	15.60 ± 1.30	0.04*
	**4**	21.50 ± 2.22	19.60 ± 1.90	0.043*
	**8**	26.80 ± 1.87	23.01 ± 1.76	0.03*
	**12**	30.10 ± 1.79	27.40 ± 1.89	0.03*
**Brunstrom Stage**	**Before treatment**	2.50 ± 0.53	2.50 ± 0.53	
	**2**	3.10 ± 0.47	2.80 ± 0.56	0.52
	**4**	3.70 ± 0.85	3.20 ± 0.57	0.73
	**8**	4.90 ± 0.57	4.00 ± 0.74	1.00
	**12**	5.60 ± 0.52	4.60 ± 0.72	0.037*
**Modified Rankin Scale**	**Before treatment**	4.00 ± 0.52	4.10 ± 0.12	
	**2**	3.60 ± 0.50	3.80 ± 0.72	1.00
	**4**	3.40 ± 0.42	3.20 ± 0.47	0.97
	**8**	2.60 ± 0.67	2.80 ± 0.79	1.00
	**12**	2.20 ± 0.70	2.60 ± 0.31	1.02

⁎ Significant difference in gains, *p* < 0.05

## Discussion

Do-It-Yourself (DIY) medical devices have become widely adopted due to their technological capability. These DIY devices open the chance to get healthcare at home [12]. Rehabilitation at home for stroke patients is becoming popular in either developed or developing countries [13]. Home rehabilitation promotes motivation leading to regular performance, which is the key to improving either physical therapy method [14]. Our DIY rehabilitation device should facilitate regular self-rehabilitation at home. During the COVID-19 pandemic era, many rehabilitation facilities have been shutting down [15]. Furthermore, stroke patients risk getting infected from COVID-19 when they leave home to get physical therapy at the rehabilitation facilities [16]. Our DIY rehabilitation device may become essential for many stroke patients to get adequate physical therapy at home during this distressing COVID-19 pandemic [17].

We also demonstrated the feasibility and safety of our DIY rehabilitation device. No complication related to the DIY rehabilitation device was found. All ten patients enrolled to use the DIY rehabilitation device can continue regular physical therapy along 12 weeks of treatment. Outcome measurement with the Brunstrom Stage of Motor Recovery (Lower extremity) and modified Rankin scale is trending more remarkable improvement with the DIY rehabilitation device than the regular rehabilitation. However, the improvement is not statistically significant within group. The results showed that the FMA-LE of experimental group was significantly higher than the control group. DIY rehabilitation device is more motivated to practice than traditional physical exercises, and it is a repetitive movement exercise that helps brain plasticity of the brain [18].

The expense for device assembly is extremely cheap as compared with other methods [18]. It cost only 30,000 Baht or 1,000 US Dollars for the materials per device. The local mechanic can easily assembly the device by following the instruction. We plan to create the video clip for device assembly and post it on the Youtube channel or the faculty website.

Our DIY rehabilitation device should be applied in patients with lower limb weakness from other causes rather than ischemic stroke, such as intracerebral hemorrhage, traumatic brain injury, multiple sclerosis, and spinal cord diseases. A clinical trial to prove the benefit of our DIY rehabilitation device is needed to approve its use in routine practice. In the meantime, the device may be a worthy option for stroke patients with lower limb weakness who have limitations to accessing the rehabilitation opportunity.

## References

[1] Tubaro M., Vranckx P., Bonnefoy-Cudraz E., Price S., Vrints C., Leys D. (2021).

[2] Muengtaweepongsa S., Dharmasaroja P., Kummark U. (2012). Outcomes of intravenous thrombolytic therapy for acute ischemic stroke with an integrated acute stroke referral network: initial experience of a community-based hospital in a developing country. J. Stroke Cerebrovasc. Dis..

[3] Palangrit S., Muengtaweepongsa S. (2015). Risk Factors of Stroke in Pathumthani Province, Thailand. J. Med. Assoc. Thail..

[4] Hendricks H.T., van Limbeek J., Geurts A.C., Zwarts M.J. (2002). Motor recovery after stroke: a systematic review of the literature. Arch. Phys. Med. Rehabil..

[5] Wandel A., Jørgensen H.S., Nakayama H., Raaschou H.O., Olsen T.S. (2000). Prediction of walking function in stroke patients with initial lower extremity paralysis: the Copenhagen stroke study. Arch. Phys. Med. Rehab..

[6] Chueluecha C. (2012). Rehabilitation in stroke. Thammasat Med. J..

[7] Raffin E., Hummel F.C. (2018). Restoring motor functions after stroke: multiple approaches and opportunities. The Neuroscientist.

[8] Sanchetee P., Sanchetee P. (2021). Ischemic Stroke.

[9] Smith T.M., Pappadis M.R., Krishnan S., Reistetter T.A. (2018). Stroke survivor and caregiver perspectives on post-stroke visual concerns and long-term consequences. Behav. Neurol..

[10] Ngamjarus C., Chongsuvivatwong V., McNeil E. (2016). n4Studies: sample size calculation for an epidemiological study on a smart device. Siriraj Med. J..

[11] Zakharov A.V., Bulanov V.A., Khivintseva E.V., Kolsanov A.V., Bushkova Y.V., Ivanova G.E. (2020). Stroke affected lower limbs rehabilitation combining virtual reality with tactile feedback. Front. Robot. AI.

[12] Stead M., Coulton P., Lindley J. (2018). Do-It-Yourself medical devices: exploring their potential futures through design fiction. Conference. Proceedings of the Design Research Society Conference.

[13] Chi N.F., Huang Y.C., Chiu H.Y., Chang H.J., Huang H.C. (2020). Systematic review and meta-analysis of home-based rehabilitation on improving physical function among home-dwelling patients with a stroke. Arch. Phys. Med. Rehab..

[14] Aidemark J., Askenäs L. (2021). A contextual understanding of IT support for physical rehab in practice: a case of “Home Rehab. Procedia Comput. Sci..

[15] Chattranukulchai P., Thongtang N., Ophascharoensuk V., Muengtaweepongsa S., Angkurawaranon C., Chalom K. (2021). An Implementation Framework for Telemedicine to Address Noncommunicable Diseases in Thailand. Asia Pac. J. Public Health.

[16] Longo E., De Campos A.C., Schiariti V. (2020). COVID-19 Pandemic: is this a good time for implementation of home programs for children's rehabilitation in low- and middle-income countries?. Phys. Occup. Ther. Pediatr..

[17] Chua J., Ong L.Y., Leow M.C. (2021). Telehealth using PoseNet-based system for in-home rehabilitation. Futur. Internet.

[18] Lo K., Stephenson M., Lockwood C. (2019). The economic cost of robotic rehabilitation for adult stroke patients: a systematic review. JBI Database Syst. Rev. Implement. Rep..

